# Effect of Soluble Solids and High Pressure Treatment on Rheological Properties of Protein Enriched Mango Puree

**DOI:** 10.3390/foods8010039

**Published:** 2019-01-21

**Authors:** Hosahalli S. Ramaswamy, Anuradha Gundurao

**Affiliations:** Department of Food Science, McGill University, Macdonald Campus, 21111 Lakeshore, Ste-Anne-de-Bellevue, PQ H9X 3V9, Canada; anuradha.gundurao@mail.mcgill.ca

**Keywords:** mango puree, whey protein, high pressure, dynamic rheology

## Abstract

The effects of high pressure treatment on the rheological properties of protein enriched mango puree were evaluated. In the first part, the original soluble solids in mango puree (28° Brix) was lowered to 26, 23 and 20° Brix, and each was supplemented with 2, 5 and 8% of whey protein to assess the influence of added protein. The samples were then evaluated by dynamic rheology. Pressure treatment resulted in a progressive increase in elastic (G′) and viscous modulii (G″) as well as complex viscosity (η*). Values of G′ were higher than G″ demonstrating that the product behaved more like a gel. Additional tests were carried out by simultaneously varying protein and soluble solids contents, and keeping the total solids at 28%. In this case, the effects of pressure levels and holding times were similar to previous results. However, the positive effects of higher protein were negated by the negative effects of lower soluble solids, resulting in an overall decreasing effect on rheology. The developed models effectively predicted the combined influence of protein and soluble solid concentrations on rheological parameters (R2 > 0.85). Sensory evaluation of 2% and 5% protein supplemented and pressure treated (500 MPa/3 min) mango puree yielded acceptable sensory qualities, resulting in a product with enriched protein content.

## 1. Introduction

Mango is one of the most consumed fruits of the tropics, with an exotic flavor, delicious taste and great color. Both ripe and unripe mangoes are used for developing many by-products. Mango puree, nectar, juice, jams, jellies and leather are some by-products of ripe mango, whereas pickles, mango powder, and salads are developed from unripe mango. There are many other products on the market like mango shakes, mango ice cream etc. Mango puree is the main source of different products that are manufactured from ripe mangoes. Mango is a rich source of β-carotene and ascorbic acid. Mango puree is rich in soluble solids and fiber, and is low in fat and proteins. From a nutrition enhancement point of view, therefore, the focus is on protein.

Whey proteins are commonly used as ingredients in many food systems because of their potential ability to form gel structures and improve the texture, flavor and stability of the product. Protein content in whey protein isolates (WPI) is typically greater than 90% and is comprised of β-lactoglobulin, α-lactalbumin, serum albumin and immunoglobulins [1]. Currently, whey protein and its different components have been commercially used in various food and pharmaceutical applications for their health benefits. Food product development applications focus both on nutritional aspects and sensory characteristics. A combination of sugar-rich mango pulp with its exotic flavor supplemented with a rich protein like WPI could produce a highly-nutritional beverage with fruit flavor.

Heat treatment has long been used for the unfolding of proteins and swelling of carbohydrates that promotes aggregation and gelatinization [2]. Browning is the major quality problem during the heat treatment of protein-based beverages. In addition, sedimentation and insolubilization are other limitations in protein beverages during processing and storage [3]. In some cases, thermal denaturation produces off flavors and/or results in the formation of toxic compounds by the destruction and cleavage of covalent bonds [4,5]. Therefore, there is a need to explore other processes to overcome these difficulties.

High pressure processing (HPP) has been proposed in many food applications, offering better quality retention compared to thermal treatment. Pressure treated fruit products retain the original color, flavor and taste of the produce. HPP has been successfully applied to various fruit products like jams, jellies and purees, as well as to commercially successful meat processing industries for a long time [6,7]. Whey protein components (α-lactalbumin and β-lactoglobulin) undergo irreversible denaturation during pressurization at 400 MPa [8,9,10]. Pressure-induced whey protein gels mainly occur during pressure holding above 400 MPa, which are also stabilized by intermolecular disulfide bonds; however, structural stability should not be influenced by pressure release [10]. An increase in pressure holding time strengthens the gel, stimulating the formation of more intensive intermolecular interactions [11,12,13]. Since HP does not affect covalent bonds, essential vitamins and nutrients are retained [6,7]. Henrichs and Rademacher (2004) [14] found that the isobaric isothermal denaturation of beta-lactoglobulin and alpha-lactalbumin follow third and second order kinetics, respectively. Lim et al. (2008) [15] found that ice cream fortified with HPP treated whey protein concentrate exhibited better overrun, foam stability and greater hardness than ice cream produced with untreated controls. Several recent studies have found the functional properties of whey and other dairy proteins to be affected by HPP [16,17,18,19,20,21,22,23].

Rheological properties of protein foods have been directly related to modifications of their functional and structural properties. Heat is the most commonly used method for the modification of functional properties. Currently, such modifications are also carried out by HPP with better acceptability [24]. The cleavage of the desired chemical bonds is controlled by the pressure that results in the desired texture and rheology of food products. Rheology of protein gels depends on solid content, pH, temperature, time and ionic strength of proteins [15,16,25]. Differences in rheological properties could be partly explained by the amount of protein incorporated into the network. The control of protein gels for various industrial applications requires a better understanding of the relationship between the structure of the aggregates and the macroscopic properties of the gel. Dynamic rheological measurements in terms of elastic modulus (solid-like characteristics) and viscous modulus (liquid-like characteristics) characterize the viscoelastic nature of protein foods during processing and gel formation. Little information is available on the effect of the addition of proteins to fruit puree subjected to HPP.

The present study deals with the characterization and modeling of rheological changes in protein enriched mango puree. Notable are the changes in dynamic rheology, as influenced by soluble solids and protein content and their model based predictions. The rheology models developed by relating the influence of product properties to rheological parameters allows for calibrated product development concepts.

## 2. Material

Mango pulp (Cv Alphanso, Cedar brand marketed by Phoenicia Products Inc. Montreal, QC, Canada) was procured from a departmental store in Montreal. The total soluble solids (TSS) of the original pulp was 28° Brix.

### 2.1. Mango Puree with Different Solids Content and Protein Concentrations

In the first part of the study, the original soluble solid level in mango puree was lowered to 20, 23 and 26° Brix by adding distilled water, and then each preparation was supplemented with WPI powder (BiPro, Davisco Foods Int., Le Sueuer, MN, USA) at 2, 5 and 8%, in order to assess the influence of added protein to mango puree at different soluble solids concentrations.

Since the addition of whey protein powder supplemented as above increased the total solids content, a second procedure was adopted to differentiate the influence of soluble solids from added protein powder. In this procedure, protein slurries were also prepared with 28% concentration so that the solid levels would be the same as those in mango puree (28° Brix). The protein slurry and mango puree were then mixed in different proportions to yield differing amounts of soluble solids and whey protein contents. The protein content selected were 2, 3, 5, 7 and 8% which simultaneously lowered the soluble solid levels in mango puree to 26, 25, 23, 21, 20%, thereby maintaining the total solids at 28%. A Pearson square rule was used to prepare these protein enriched mango purees.

### 2.2. High Pressure Equipment and Treatment

A semi-continuous-type pressure vessel (10 cm diameter and 55 cm height) with stainless steel cylindrical pressure chamber was used for this study (model ACIP 6500-5-12VB, ACB, Nantes, France). The maximum operating pressure level of the equipment was 650 MPa. The pressure come up time varied between 1 and 2 min, with pressures varying between 425 and 575 MPa. The depressurization time was less than 20 s. Samples were submerged in the pressure chamber containing water as the hydrostatic fluid medium. The treatment temperature was maintained between 20–25 °C. Due to compression, the medium and sample temperatures increase about 3 °C per 100 MPa raise in pressure level. Hence, the initial temperature of medium and sample were lowered accordingly before treatment. A thermocouple inside the chamber, attached to a temperature logger, was used to measure the temperature of the medium during pressure treatment.

For the first part of the study, protein supplemented mango puree at each soluble solids concentration was subjected to three pressure levels and three holding times (425, 500 and 575 MPa and 0, 3 and 6 min holding times). In the second part, the samples were prepared with different soluble solids and protein contents, but with total solids maintained at 28%. They were pressure treated at five levels of pressure (425, 450, 500, 550, 575 MPa) and holding times (0, 1, 3, 5, 6 min). Finally, selected protein enriched formulations were pressure treated at 500 MPa with a 3 min holding time for sensory evaluation studies.

### 2.3. Experimental Design

A three level factorial design was selected to study the main effect and interaction effects in the first part of the study. For the second part, the experimental design adopted was a modification of Box’s central composite design for three variables at five levels each. The three independent variables were protein concentration (X_1_), pressure (X_2_) and holding time (X_3_). The independent variable coded values were −1.5 (lowest level), −1, 0 (middle level), 1 and 1.5 (highest level). The pressure and soluble solids were co-variables (one determining the complementary level of the other, totaling to 28%). The actual values and the corresponding coded values of the three independent variables and responses of dependent variables are shown in Table 1. The correspondence between the coded and actual values can be obtained using the following formula:

(1) $$Z=\left(\frac{X-X^{o}}{\Delta X}\right)$$

where Z is the coded value, X is the corresponding actual value, X° is the actual value in the center of the domain, and ΔX is the increment of X corresponding to 1 unit of Z.

The complete design consisted of 24 experimental points including six replications of the center point to estimate the pure error of the analysis and to predict the lack of fit of the models. It was assumed that the three independent variables affected each of three dependent Y variables (responses). Responses under observations were: elastic modulus (Y_1_), viscous modulus (Y_2_) and complex viscosity (Y_3_). Results were analyzed to compare experimental values with model predictions.

### 2.4. Statistical Analyses

Experimental data were analyzed to fit the 2nd order polynomial equation to all dependent Y variables:

(2) $$\begin{array}{l} Y=a_{0+}a_{1}X_{1}+a_{2}X_{2}+a_{3}X_{3}+a_{12}X_{1}X_{2}+a_{13}X_{1}X_{3}+a_{23}X_{2}X_{3}+\\ a_{11}X_{1}{}^{2}+a_{22}X_{2}{}^{2}+a_{33}X_{3}{}^{2}+a_{123}X_{1}X_{2}X_{3} \end{array}$$

where a_n_ are constant regression coefficients and X_1_ (protein concentration), X_2_ (pressure level), and X_3_ (holding time) are coded independent variables.

SAS software Stat 8.0 (SAS Institute, 1999, Cary, NC, USA) was used to perform stepwise procedure to simplify the models and to obtain analyses of variance and regression coefficients.

### 2.5. Rheological Measurements

A controlled-stress rheometer (AR 2000, TA Instruments, New Castle, DE, USA) equipped with a computer control software (Rheology Advantage Data Analysis Program, TA, New Castle, DE, USA) was used to study dynamic oscillatory measurement of the mango puree sample. A 60 mm parallel plate attachment was used with a gap of 1000 microns. The AR 2000 was supplemented with an efficient Peltier temperature control system, and the sample temperatures were precisely controlled and monitored. For each test, a measured volume (approximately 2 mL) of mixed samples was placed on the bottom plate of the rheometer. The test temperature was maintained at 25 °C. Dynamic oscillatory tests were carried out at a frequency sweep from 0.1 to 10 Hz. The oscillation stress was selected based on linear part of the viscoelastic range (0.1–0.2 Pa). Each time a new sample was used for rheological measurement. The elastic modulus (G′), viscous modulus (G″) and complex viscosity (η*) were obtained directly from the software (Rheology Advantage, TA version 2.3, New Castle, DE, USA).

### 2.6. Sensory Evaluation of HP Treated Protein Enriched Mango Puree

The sensory evaluation of mango puree with different protein concentrations (2, 5 and 8%) before and after high pressure treatment (500 MPa & 3 min) was performed using a nine point hedonic scale by a panel of ten judges with an equal ratio of men and women. The quality parameters were color, sweetness, mouth feel, flavor, and acceptability. All samples were served in closed, opaque cups to retain flavor until the test was done. Samples were served in randomized order to avoid any effects based on the serving order. The judges were asked to compare protein added samples with each other and with pure untreated mango puree (control).

## 3. Results and Discussion

### 3.1. HPP Effects on Protein Supplemented Mango Puree with Different Soluble Solids Content

#### 3.1.1. Effect of Treatment Time

**Figure 1:**
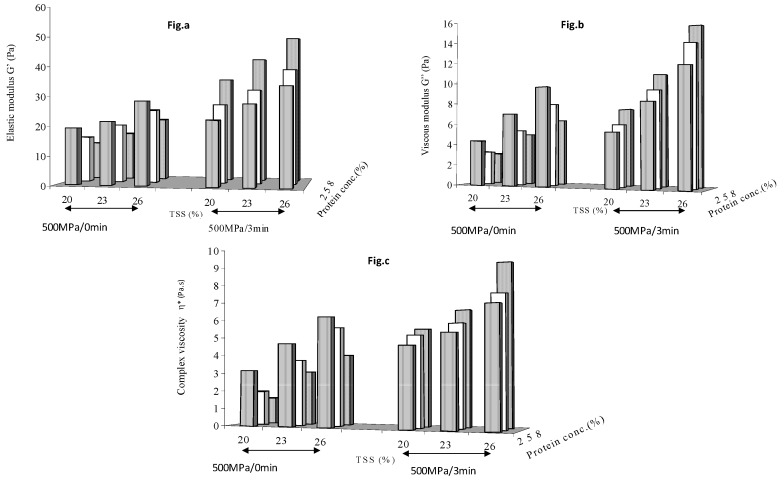
Effect of holding time (zero and 3 min) at 500 MPa on (**a**) elastic modulus (**b**) viscous modulus and (**c**) complex viscosity of protein enriched mango puree.

Figure 1 shows the effect of holding time (0 and 3min) at HP treated at 500 MPa on dynamic modulii of mango puree. An increase in soluble solid contents of mango puree increased all rheological parameters at each of the supplemented protein concentration levels. The pressure pulse treatment (zero min holding time) did not have any influence on the added protein, and G′, G″ and η* of the protein enriched samples decreased as result of an increase in protein concentration. Since there was no pressure effect and protein constituted an inactive bulk, higher protein concentrations resulted in a lowering of the viscoelastic properties of mango puree. However, with a 3 min holding time, the pressure treatment caused a significant (*p* < 0.05) increase in the viscoelastic property values at each soluble solid content level and at each protein concentration. Soluble solids levels had a more dominant effect than protein concentration in increasing the viscoelastic parametric values of pressure treated, protein enriched mango puree. At any chosen pressure level, a minimum holding time of 3 min was essential for the rheology build-up of protein enriched samples.

**Figure 2:**
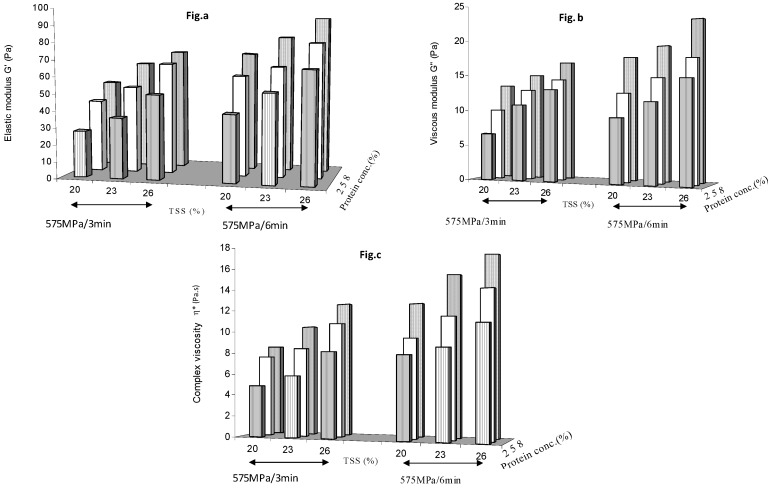
Effect of holding time (3 and 6 min) at 575 MPa on (**a**) elastic modulus (**b**) viscous modulus and (**c**) complex viscosity of protein added mango puree.

Figure 2 shows the effect of holding time (3 and 6 min) on G′, G″ and η* at 575 MPa. A clear increase in rheological parameters was observed at each protein concentration and soluble solids concentration levels as the holding time increased from 3 min to 6 min. This supports the earlier observation that a minimum holding time of 3 min was required at 500 MPa to initiate the denaturation or texturization process, and the 6 min treatment further reinforced the gel network formation.

#### 3.1.2. Effect of Pressure Level

**Figure 3:**
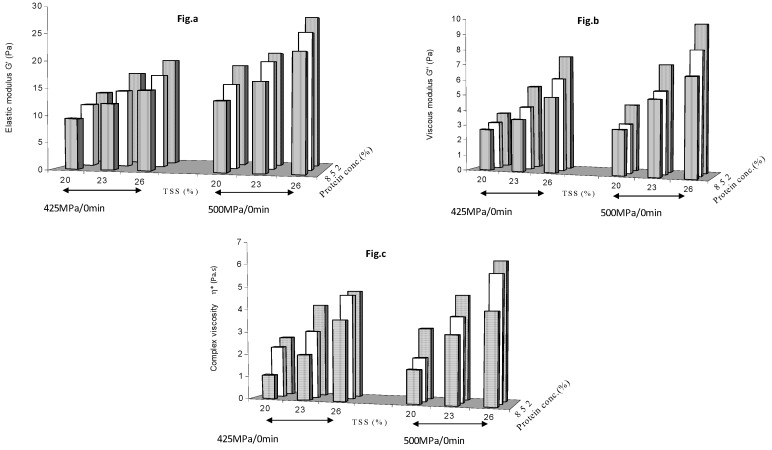
Effect of pressure (425 and 500 MPa) at zero min holding time on (**a**) elastic modulus (**b**) viscous modulus and (**c**) complex viscosity of protein added mango puree.

**Figure 4:**
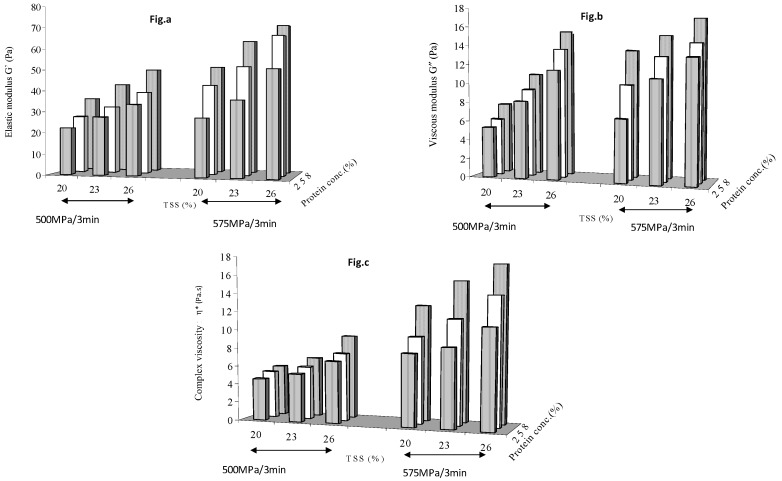
Effect of pressure (500 and 575 MPa) at 3 min holding time on (**a**) elastic modulus (**b**) viscous modulus and (**c**) complex viscosity.

This section shows the effect of pressure level on rheological properties of protein added mango puree between (1) 425 and 500 MPa with a holding time of 0 min (Figure 3) and (2) 500 and 575 MPa with a holding time of 3 min (Figure 4). The zero min holding time results at 425 and 500 MPa (Figure 3) were similar to that previously observed at 500 MPa (Figure 1). The protein effect curves appear different, but this is caused by the positional reversal of pressure level scale. Results observed at 3 min holding time and pressure levels of 500 and 575 MPa (Figure 4) were again similar to those observed previously (Figure 2), with both soluble solids and protein concentrations contributing to gel structure build up as result of HP treatment. G′, G″ and η* values increased when pressure was increased from 425 to 500 to 575 MPa. The values of G′ varied between 9.33 Pa at 425 MPa/0 min and 93.3 at 575 MPa/6 min, G″ from 2.69 Pa and 22.8 whereas η* varied from 1.061 and 17.2 Pa.s.

Holding time had a more significant effect (*p* < 0.05) than applied pressure for formation of stabilizing interactions linking the network and to increase the gel strength. The values of elastic modulus (G′) were higher than those of viscous components (G″) throughout the frequency range. This shows that the gelling behavior of protein has an effect of both pressure and holding time greater than zero. The elastic component predominated over viscous components, indicating the viscoelastic behavior of mango puree for all the protein concentrations.

### 3.2. HPP Effects on Protein Supplemented Mango Puree with Same Total Solids Content

The addition of protein increases the solid levels and simultaneously decreases the moisture content in mango puree. Thus, the focus of the second part of this study was to vary the protein level in mango puree maintaining the constant total solids and moisture levels.

#### Effect of Protein Concentration, Pressure and Time on G′, G″ and H*

**Figure 5:**
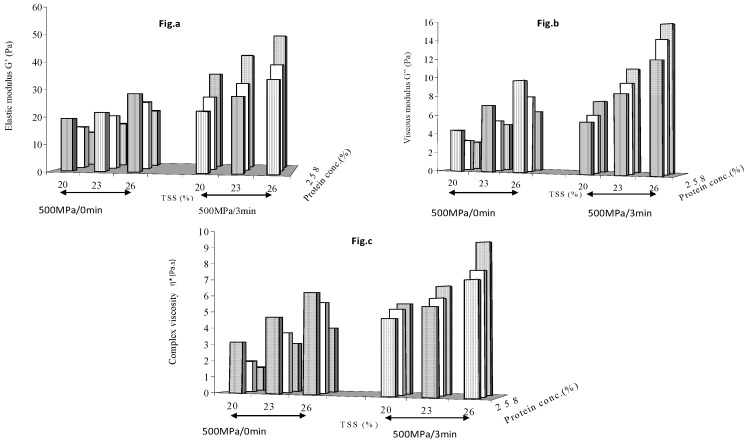
Elastic modulus as influenced by protein concentration and (**a**) pressure (**b**) holding time.

**Figure 6:**
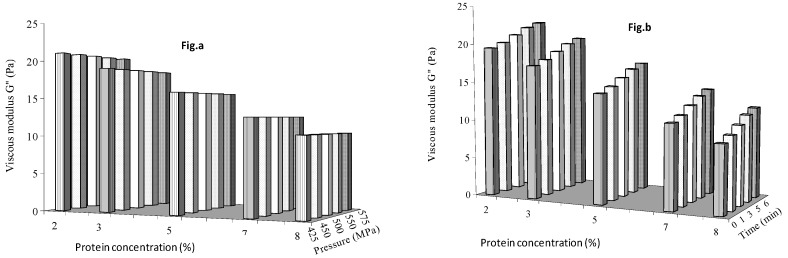
Viscous modulus as influenced by protein concentration and (**a**) pressure (**b**) holding time.

**Figure 7:**
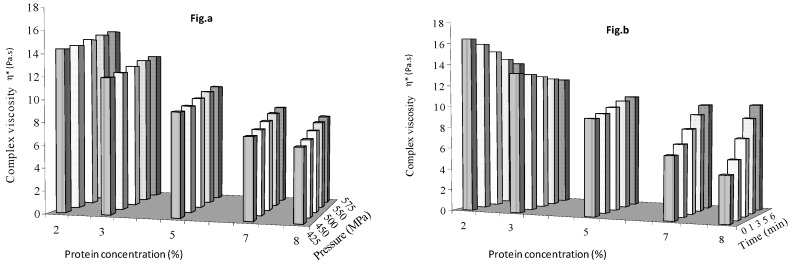
Effect of protein concentration (**a**) pressure and (**b**) holding time on complex viscosity of mango puree.

In qualitative terms, the oscillatory rheology curves provide a fingerprint of the state of the microstructure, and thus, characterize the viscoelastic properties of fluid foods. Figure 5a shows the effect of protein concentration and pressure with a 3 min treatment time, and Figure 5b shows the effect of protein concentration and time on G′ of mango puree at the mid-level pressure of 500 MPa. Similar results for viscous modulus and complex viscosity are shown in Figure 6 and Figure 7, respectively. G′ decreased with protein concentration (and the simultaneous decrease in solids level in mango puree). Thus, changes in solid contents of mango puree had a greater effect on G′ than protein concentrations at all other chosen pressure and time levels. Pressure had positive effect on elastic modulus, but a decreasing trend was observed with holding times up to 5%, with protein concentrations reversing to an increasing trend at 7 and 8%. This indicated that a minimum of 5% protein concentration was necessary to increase G′ of mango puree with holding time.

Viscous modulus did not show significant change with pressure at the chosen protein concentration levels (Figure 6a), but increased with holding time (Figure 6b). G″ showed similar effects with protein concentration as G′. Complex viscosity showed an increasing trend with pressure but decreased with protein concentration levels (Figure 7a). Similar to G′, η* decreased with holding time at 2 and 3% protein concentration but increased thereafter at 5, 7 and 8% (Figure 7b). So, protein concentration >3% would increase the η* of mango puree.

### 3.3. Model Development

A second order polynomial equation (2) was fitted to the experimental data (Table 1) of G′, G″ and η* to evaluate the effect of protein concentration, pressure and time, using RSREG procedure of SAS. The best equation was chosen from stepwise selection testing the adequacy and fitness by analysis of variance (ANOVA). The ANOVA results for each of the dependent variables with coefficient of determinations (R^2^) are presented in Table 2. The models developed for G′, G″ and η* seemed adequate with significant F values and non-significant lack of fit, but it showed R^2^ < 0.80, considering the low percentage of variability explained.

The polynomial equations for the responses are shown below:

G′ = 62.2 − 15.9 X_1_ + 6.6006 X_1_*X_2_ + 8.16 X_1_*X_3_ + 6.41 X_1_*X_2_*X_3_ (3)

G″ = 15.6 − 3.46 X_1_ + 1.26 X_1_*X_3_ (4)

η* = 10.2 − 2.601 X_1_ + 1.0608 X_1_*X_2_ + 1.32 X_1_*X_3_ + 1.036 X_1_*X_2_*X_3_ (5)

where X_1_ is protein concentration, X_2_ is pressure and X_3_ is time.

The independent effects of protein content and soluble solids concentration were computed from the above models, keeping in mind that an increase in protein concentration also means a decrease in soluble solids concentration. For example, the central point value is a result of 5% protein and 23% soluble solids. The formulation with 3% protein will have a higher soluble solids of 25%. To get the value for 3% protein and 23% soluble solids from a model value at 3% protein (and 25% soluble solids), the contribution from excess 2% soluble solids need to be deducted. Similarly, to get the value for 7% protein at 23% soluble solids to the model value at 7% protein (and 21% soluble solids) from the central value, the effect of 2% soluble solid concentration addition needs to be supplemented. A similar approach can be used for other protein and soluble solid concentrations used in the study.

### 3.4. Independent Effects of Protein Concentration and Soluble Solids

**Figure 8:**
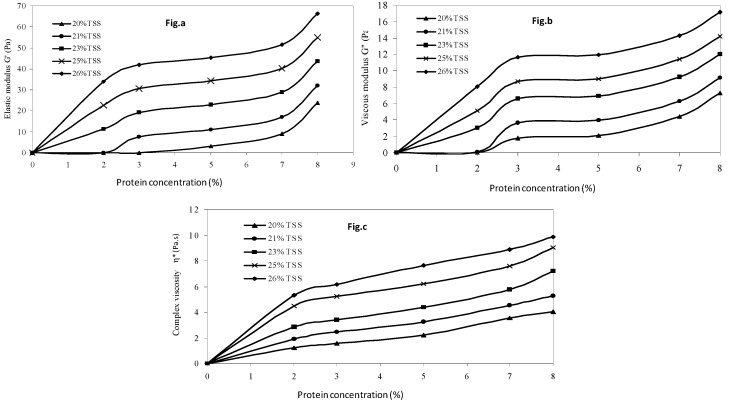
Predicted individual effects of soluble solids and protein concentration on (**a**) elastic modulus, (**b**) viscous modulus and (**c**) complex viscosity at 500 MPa and 3 min holding time.

**Figure 9:**
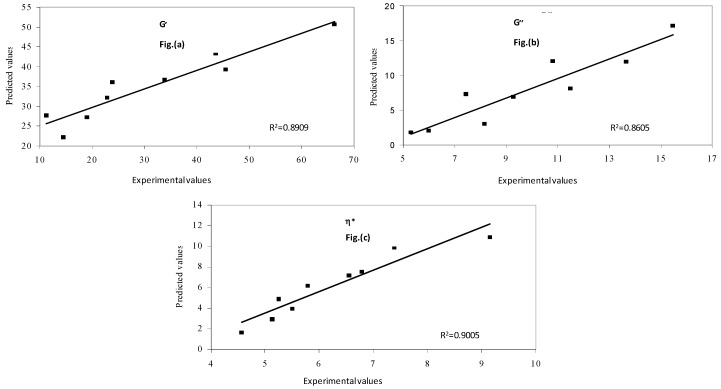
Predicted and experimental values of (**a**) elastic modulus (**b**) viscous modulus and (**c**) complex viscosity at 500 MPa and 3 min holding time.

The effect of protein concentration and soluble solid levels in mango puree were isolated using the hypothetical model, as explained in the previous section, for pressure treatment at 500 MPa and 3 min holding time. An increase in rheological parameters was observed with the protein concentration at each soluble solid content level (Figure 8). The soluble solids clearly over-dominated the effect of protein concentration. The developed models helped in predicting the effect of different ingredients on rheological parameters. A good correlation was found between the predicted values and combined effect of protein and soluble solids concentration (R^2^ > 0.85), as represented in Figure 9.

### 3.5. Sensory Evaluation of HP Treated Protein Enriched Mango Puree

#### 3.5.1. Comparison of Protein Added Samples

Significant (*p* < 0.05) change in color was visible in the sample enriched with 8% protein after pressure treatment, whereas mango puree with 2% and 5% protein concentrations showed insignificant changes (Table 3). The color was acceptable before and after pressure treatment. Sweetness was not affected by pressure, but the 5% protein added sample was preferred to the 2% and 8% samples. Mouth feel also increased significantly (*p* < 0.05), favoring samples which had undergone pressure treatment. Flavor remained the same before and after pressure treatment. However, mango puree with 8% protein concentration showed slight off flavor and bitterness, but not at 2 and 5%. Overall acceptance was better for the 2 and 5% protein added samples than the 8% sample.

#### 3.5.2. Comparison of Control with Protein Added Samples

An insignificant (*p* < 0.05) change in color between control and protein added mango puree was noticed before pressure treatment, whereas change was significantly visible after treatment. Sweetness reduced with protein concentration, and the sample with 5% protein was preferred to the 2%, 8% and control samples. Mouth feel was similar for control and protein added samples. Control had strong flavor, and a significant change (*p* < 0.05) was noticed only with 8% protein concentration.

## 4. Conclusions

Soluble solids have a major effect on rheological parameters compared to added protein. The effects of pressure and holding time on protein added mango puree were similar during the first and second part of the experiment. An increase in pressure increased G′, G″ and η*. Values of G′ were higher than G″, exhibiting the gelling properties of product. However, in the first part, the soluble solid contents of mango puree and protein concentration showed positive effects on G′, G″ and η *, except when the treatment was given as a pressure pulse (zero holding time). More work is needed to fully explain the pulse pressure effect on protein added mango puree. In the second part, the effect of protein concentration effect was dominated by a simultaneous decrease in soluble solid contents, resulting in an overall decrease in rheological parameters with protein content. G″ increased with added protein, whereas a minimum of 5% protein concentration was required to observe an increasing trend of G′ and η*. The isolated effect of protein from soluble solids content showed its gelling behavior. The models effectively predicted the rheological parameters influenced by soluble solid contents and protein combinations. Sensory evaluation results indicated that protein concentrations up to 5% are preferred compared to 8%. High pressure treatment did not help in reducing the off flavor at higher protein concentrations.

## Figures and Tables

**Table 1:** 3-variable, 5-level central composite design and responses of dependent variables to the dynamic rheological properties of pressure treated protein enriched mango puree.

Run	Independent Variables ^a^ (Coded and Real Values)			Dependent Variables ^b^		
	X_1_	X_2_	X_3_	Y_1_	Y_2_	Y_3_
1	3 (−1)	450 (−1)	1 (−1)	14.63	20.61	89.60
2	3 (−1)	450 (−1)	5 (1)	12.84	17.92	78.64
3	3 (−1)	550 (1)	1 (−1)	16.08	22.26	98.57
4	3 (−1)	550 (1)	5 (1)	12.56	19.06	76.61
5	7 (1)	450 (−1)	1 (−1)	7.453	11.74	45.33
6	7 (1)	450 (−1)	5 (1)	7.279	11.63	44.24
7	7 (1)	550 (1)	1 (−1)	7.484	11.83	45.51
8	7 (1)	550 (1)	5 (1)	12.19	17.22	74.66
9	5 (0)	500 (0)	0 (−1.5)	7.667	11.32	46.83
10	5 (0)	500 (0)	6 (1.5)	11.31	15.47	69.37
11	2 (−1.5)	500 (0)	3 (0)	16.24	22.59	99.51
12	2 (−1.5)	500 (0)	5 (1)	12.48	19.89	75.87
13	5 (0)	575 (1.5)	3 (0)	10.95	16.92	66.72
14	5 (0)	575 (1.5)	5 (1)	11.3	15.32	69.31
15	8 (1.5)	500 (0)	3 (0)	7.718	11.92	47.01
16	8 (1.5)	500 (0)	1 (1)	7.911	11.98	48.24
17	5 (0)	425(−1.5)	3 (0)	9.811	14.17	60.00
18	5 (0)	425 (−1.5)	5 (1)	8.837	12.49	54.11
19(6X)	5 (0)	500 (0)	3 (0)	9.971	14.93	60.85

^a^*X*_1_, Protein concentration (%); *X*_2_, Pressure applied (MPa) and *X*_3_, Holding time (min). ^b^*Y*_1_, Complex viscosity (Pa.s); *Y*_2_, Viscous modulus (Pa), *Y*_3_, Elastic modulus (Pa).

**Table 2:** Regression coefficient and analysis of variance for six response variables.

Coefficient	Complex Viscosity	Viscous Modulus	Elastic Modulus
a_0_	10.2 ***	15.6 ***	62.2 ***
Linear			
a_1_	−2.601 ***	−3.46 ***	−15.9 ***
a_2_	0.40609	0.423	3.29
a_3_	0.512	0.4204	-0.24
Interactions			
a_12_	1.0608 **	0.361	6.6006 **
a_13_	1.32 ***	1.26 *	8.16 ***
a_23_	0.311	0.377	1.92
a_123_ a_11_ a_22_ a_33_	1.036 * 0.8024 0.359 0.0516	0.391 0.4069 0.112 0.0962	6.41 * 4.19 2.25 0.39
%Variability explained (R^2^)	0.79	0.65	0.79
F-value			
Regression	18.08	21.10	18.04
Lack of fit	2.76	1.85	2.83
Probability of F			
Regression	<0.0001	0.0001	<0.0001
Lack of fit	N. S.	N. S.	N. S.

*** Highly significant (*p* < 0.01), ** Significant (*p* < 0.05), * Borderline significant (0.05–0.1).

**Table 3:** Results of sensory evaluation of control and protein enriched mango puree before and after pressure treatment (higher values represent better acceptability) (B_PT_-before pressure treatment; A_PT_-after pressure treatment).

	Control	2%PC		5%PC		8%PC	
		B_PT_	A_PT_	B_PT_	A_PT_	B_PT_	A_PT_
Color	8	8	8	8	7	8	5
Sweetness	7	7	7	8	8	5	5
Flavor	8	7	7	6	6	5	5
Mouth feel	7	7	8	6	8	5	7
Acceptability	8	8	8	7	6	5	5
